# High-cholesterol diet does not alter gut microbiota composition in mice

**DOI:** 10.1186/s12986-017-0170-x

**Published:** 2017-02-16

**Authors:** Lidiya G. Dimova, Nikola Zlatkov, Henkjan J. Verkade, Bernt Eric Uhlin, Uwe J. F. Tietge

**Affiliations:** 1 Department of Pediatrics, University Medical Center Groningen, University of Groningen, Groningen, The Netherlands; 2 Laboratory for Molecular Infection Medicine Sweden (MIMS) and Department of Molecular Biology, Umeå University, Umeå, Sweden

**Keywords:** Diet, Cholesterol, microbiota, *Ldlr*-knockout, Bile, Neutral sterols

## Abstract

**Introduction:**

Western diet containing both saturated fat and cholesterol impairs cardio-metabolic health partly by modulating diversity and function of the microbiota. While diet containing only high fat has comparable effects, it is unclear how diets only enriched in cholesterol impact the microbiota. Therefore, we aimed to characterize the response of host and microbiota to a high cholesterol (HC) diet in mice susceptible to cardio-metabolic disease.

**Methods:**

LDLR knockout mice received either 1.25% HC or no cholesterol containing control diet (NC) for 12 weeks before characterizing host cholesterol metabolism and intestinal microbiota composition (next generation sequencing).

**Results:**

HC diet substantially increased plasma (1.6-fold) and liver cholesterol levels (21-fold), biliary cholesterol secretion (4.5-fold) and fecal neutral sterol excretion (68-fold, each *p* < 0.001) but not fecal bile acid excretion. Interestingly, despite the profound changes in intestinal cholesterol homeostasis no differences in microbial composition between control and HC-fed mice were detected. In both groups the main phyla were *Bacteroidetes* (55%), *Firmicutes* (27%) and *Verrucomicrobia* (14%).

**Conclusion:**

Our results demonstrate that in mice HC diet alone does not alter the microbiota composition despite inducing substantial adaptive changes in whole body cholesterol homeostasis. The impact of Western diet on intestinal microbiota thus appears to be mediated exclusively by its high fat content.

**Electronic supplementary material:**

The online version of this article (doi:10.1186/s12986-017-0170-x) contains supplementary material, which is available to authorized users.

## Introduction

The intestinal microbiota can exert profound effects on the metabolism of the host. Changes in gut community structure and function have been implied in metabolic syndrome-related conditions such as obesity [1, 2], type 2 diabetes [3] and atherosclerosis [4, 5]. Diet emerges as an important factor influencing microbiota composition since nutrient abundance can promote the growth of different bacterial groups by affecting bacterial metabolism and adaptability [6]. The shift from a low-fat plant-polysaccharide-rich diet towards a high-fat Western style diet has been shown to strongly modify the gut microbiota within days of administration [7] by increasing the representatives of *Mollicutes*, *Erysipelotrichi* and *Bacilli* classes of *Firmicutes* along the intestinal axis [8]. These changes conceivably contribute to metabolic disease, since the predominance of *Firmicutes* over *Bacteroidetes* has been associated with obesity and metabolic syndrome in both mice [9] and humans [10]. High fat diet has effects similar to Western diet on the gut ecosystem [11] resulting in an altered metabolomic signature of dominant phylotypes as *Bacteroidetes* [8] although this has not been unequivocally established and the diet effect might depend on additional factors such as the choice of model [12]. However, in addition to high fat, Western diet also contains high levels of dietary cholesterol, leading to an increase in LDL cholesterol, the main risk factor for cardiovascular disease development. Mice lacking the intestinal cholesterol transporter Npc1l1 were shown to develop alterations in their gut microbiota compared to wild type mice [13]. Whether such changes are due to an increased cholesterol abundance in the intestine has thus far not been determined. Here, we aimed to evaluate the impact of an exclusive increase in dietary cholesterol on whole body cholesterol homeostasis as well as on the gut microbiome in *Ldl*-receptor knockout mice. These mice were chosen since they represent a widely used model for several aspects of cardio-metabolic disease such as atherosclerosis [14] or non-alcoholic steatohepatitis [15], pathologies in which changes in microbiota composition have been mechanistically implicated [4, 5, 16, 17]. Our results demonstrate that the composition of the intestinal microbiota remained remarkably stable despite substantial changes occurring in overall cholesterol metabolism in response to the HC diet. These data furthermore imply that the impact of Western diet on the microbiota is mediated by its high fat rather than its high cholesterol content.

## Materials and methods

### Animals and diets

Male B6.129S7-*Ldlr* ^*tm1Her*^ mice (Jackson Laboratories, Bay Harbor, Maine, USA) were bred in our facility. To avoid confounding effects of kinship, the selected animals included in this experiment were littermates. After weaning they were individually housed under temperature controlled conditions with 12 h light/dark cycles. Mice were maintained on semisynthetic AIN93G diet (D10012G, Research Diets) until 12 weeks of age when half of them were switched to a 1.25%-cholesterol containing re-formulation of the same diet (D12110502, Research Diets comparable to previous work [18]), which was then continued for an additional 12 weeks. Food and water were provided *ad libitum.* All animal experiments were approved by the Animal Care and Use Committee at the University of Groningen, The Netherlands.

### Assessment of host cholesterol metabolism

Blood was collected by heart puncture and placed on ice. Plasma was collected after centrifugation at 3000 rpm for 10 min at 4 °C and was used for colorimetric quantification of total plasma cholesterol using a commercially available kit (Roche, Mannheim, Germany). For the determination of hepatic cholesterol and triglyceride content, 300 mg of frozen tissue were used for lipid extraction with the Bligh and Dyer method. Lipids were dissolved at 37 °C in 0.1% Triton-X100 in H_2_O and quantified with commercially available kits (Roche, Mannheim, Germany). Bile was continuously collected for 30 min after biliary duct cannulation [19]. Cholesterol in the bile was measured by gas chromatography after lipid extraction using the general procedure of Bligh and Dyer as described [20]. Bile acids in the bile were quantified using a fluorometric assay as published [20]. For the determination of fecal neutral sterols and bile acids, 50 mg of feces were saponified, followed by separation of neutral and acidic sterols by triple petroleum ether extraction [21]. The organic phase containing the neutral sterols, was processed as for determination of biliary cholesterol. Total bile acids were extracted from the aqueous phase using a SepPak-18 column, methylated and measured by gas chromatography [20].

### Microbial community analysis

DNA was extracted from cecum contents using the MoBio PowerFecal DNA extraction kit. The microbial 16S rRNA gene was amplified with barcoded universal 341 F-785R primers and the sequencing of the corresponding products was performed at 300 bp paired-end read with Illumina MiSeq V3 (LGC Genomics, Berlin, Germany) to a total of 1 million read pairs. Demultiplexing of all samples was done using Illumina’s CASAVA data analysis software. Reads with lower than 100 bp were discarded. 16S pre-processing and operational taxonomic unit (OTU) picking from amplicons was carried out with Mothur 1.33 using the 16S Silva reference alignment. The OTU picking by clustering was set at 97% identity level using the cluster split method. *De novo* phylogenetic tree generation was performed with the FastTree method. Singleton OTUs were excluded from the analysis, as were OTUs with a relative abundance lower than 0.01%. The taxonomical assignment of the OTUs and the calculations for *α* and *β* diversity were executed with the QIIME pipeline*.* We used UniFrac to determine which of the microbial communities represented in our mice were significantly different, as well as the basis for a distance matrix to cluster the samples using Unweighted Pair Group Method with Arithmetic Mean (UPGMA ) and to perform principal component analysis.

### Gene expression

Gene expression analysis was performed using quantitative real-time PCR. After sacrifice the small intestine was dissected and washed with PBS in the presence of a protease inhibitor cocktail (cOmplete, Roche, Almere, Netherlands). The tissue was snap-frozen and stored at −80 °C until analysis. RNA was isolated using Trizol reagent following the manufacturer’s instructions (Life Technologies, Thermo Fischer Scientific). RNA was quantified with NanoDrop 2000 (Life Technologies, Thermo Fischer Scientific) and a total of 1 μg was used for cDNA synthesis with M-MLV reverse transcriptase (Sigma). Real time PCR was performed on a StepOne RealTime PCR instrument (Applied Biosystems). Target gene expression levels were normalized to the expression of the acidic ribosomal phosphoprotein *36b4*.

### Statistical analysis

The differences between physiological parameters in the groups were compared with the Mann–Whitney U-test. Analysis of similarities was used to examine clustering of microbial communities using the unweighted UniFrac distance matrices. QIIME v 1.8.0 and R v3.3.0 with the packages phyloseq 1.4.5, cluster 1.14.4 and ggplot2 0.9.3.1 were used for statistics and graphics. Analyses at phylum and genus level were performed by aggregating all OTUs which had identical classification at the given taxonomic level, and normalizing samples by their total abundance. We omitted the unclassified taxa and taxa with mean relative abundance lower than 0.01% since the sample size was relatively small and the analysis focused on taxa that were expressed in most samples in order to get biologically meaningful results. The statistical significance threshold we used was an alpha of 0.05.

## Results and discussion

### High dietary cholesterol intake substantially changes overall cholesterol metabolism

The addition of 1.25% cholesterol to a low fat semisynthetic diet for 12 weeks did not induce changes in body weight compared to the control diet with no added cholesterol (NC) (Table 1). However, mice fed the high-cholesterol (HC) diet developed elevated plasma cholesterol levels compared to control mice maintained on the NC diet (+39%, *p* < 0.01) (Table 1). In addition, the HC group had an increased hepatic content of cholesteryl esters (+96%, *p* < 0.01) as well as triglycerides (+63%, *p* < 0.01). These data indicate, consistent with previous findings, that feeding a diet enriched in cholesterol results in cholesterol accumulation in plasma and tissues of *Ldlr-*knockout mice [22]. The mRNA expression of *Npc1l1* in the proximal small intestine, the main transporter involved in cholesterol absorption, was not significantly different between both experimental groups (Additional file 1: Figure S1).

**Table 1:** Metabolic changes in Ldlr-knockout mice after 12 weeks high-cholesterol diet feeding

	Control (*n* = 4–6)	High-cholesterol (*n* = 5–8)	Significance
Metabolic parameter			
Body weight (g)	26.91 (24.38 to 28.04)	27.07 (24.87 to 30.06)	n.s.
Total plasma cholesterol (mmol/L)	10.55 (8.585 to 11.26)	17.27 (13.64 to 22.93)	*p* < 0.01
Hepatic cholesterol (mmol/g)	0.60 (0.25 to 5.59)	12.60 (7.67 to 14.27)	*p* < 0.01
Hepatic triglycerides (mmol/g)	1.51 (0.45 to 2.88)	4.05 (3.34 to 4.17)	*p* < 0.01
Cholesterol balance (μmol/day/100 g BW)			
Biliary bile acids	32.89 (25.23 to 51.87)	35.21 (22.15 to 61.75)	n.s.
Fecal bile acid (total) excretion	4.64 (3.74 to 16.55)	15.22 (2.59 to 22.69)	n.s.
Biliary cholesterol secretion	7.76 (2.44 to 9.31)	34.94 (14.58 to 55.13)	*p* < 0.001
Fecal neutral sterols excretion	6.56 (3.97 to 9.47)	448.5 (384.6 to 558.7)	*p* < 0.001
Dietary cholesterol	2.93 (2.359 to 2.949)	439.6 (394.4 to 522.8)	*p* < 0.001
Primary and secondary bile acids in feces (μmol/day/100 g BW)			
Allo-cholic acid	0.16 (0.09 to 1.28)	0.70 (0.06 to 1.08)	n.s.
Alpha-muricholic acid	0.86 (0.60 to 2.50)	3.06 (0.21 to 7.25)	n.s.
Deoxycholic acid	0.97 (0.30 to 3.98)	2.92 (0.12 to 6.33)	n.s.
Cholic acid	2.86 (0.53 to 5.17)	2.05 (0.06 to 5.98)	n.s.
Chenodeoxycholic acid	0.29 (0.08 to 0.48)	0.36 (0.04 to 0.59)	n.s.
Hyodeoxycholic acid	0.10 (0.03 to 0.19)	0.15 (0.05 to 0.40)	n.s.
Ursodeoxycholic acid	0.10 (0.02 to 0.17)	0.16 (0.05 to 0.50)	n.s.
Beta-muricholic acid	0.68 (0.17 to 1.69)	2.58 (0.05 to 10.07)	n.s.
Omega-muricholic acid	1.18 (0.41 to 3.53)	1.74 (0.09 to 3.53)	n.s.

Data are given as median and range. Statistical significance was tested with Mann–Whitney U-test or two-way ANOVA post-hoc Bonferoni test

Cholesterol can either be cleared directly from the systemic circulation [23] or by prior conversion to bile acids [24]. HC feeding resulted in a 4.5-fold (*p* < 0.001, Table 1) increase in biliary cholesterol secretion and a 68-times higher fecal neutral sterol excretion (*p* < 0.001). On the other hand, both biliary bile acid secretion and fecal bile acid excretion remained comparable between the groups. However, certain microbial taxa are involved in bile acid metabolism and can thereby substantially affect the composition of fecal bile acids [25]. Primary bile acids synthesized from cholesterol in hepatocytes reach the intestinal lumen via the bile. In the large intestine unabsorbed bile acids are first deconjugated by the bacterial enzyme bile salt hydrolase; next they are dehydroxylated by bacteria to form the secondary bile acids [26]. Thus, changes in the fecal bile acid composition can provide important cues for an altered microbiota function. However, HC diet did not induce any appreciable change in the distribution of fecal secondary bile acids when compared to the NC group (Table 1). The results also indicate that the HC diet did not induce adaptations in microbiota metabolism with respect to bile acid conversion. However, these data still leave the possibility that dietary HC critically affected overall microbiota composition.

### HC diet does not change cecal microbiota composition

We next applied a deep sequencing approach to obtain an in depth characterization of the microbiota composition in the HC compared with the NC group. We first compared the relative abundance of each bacterial group and then calculated the ratio between *Firmicutes* and *Bacteroidetes* and performed analyses of the 8 most abundant classes. After setting the exclusion criteria for low abundance to 0.01% a total of 683 OTUs were included in the subsequent analysis.

**Fig. 1:**
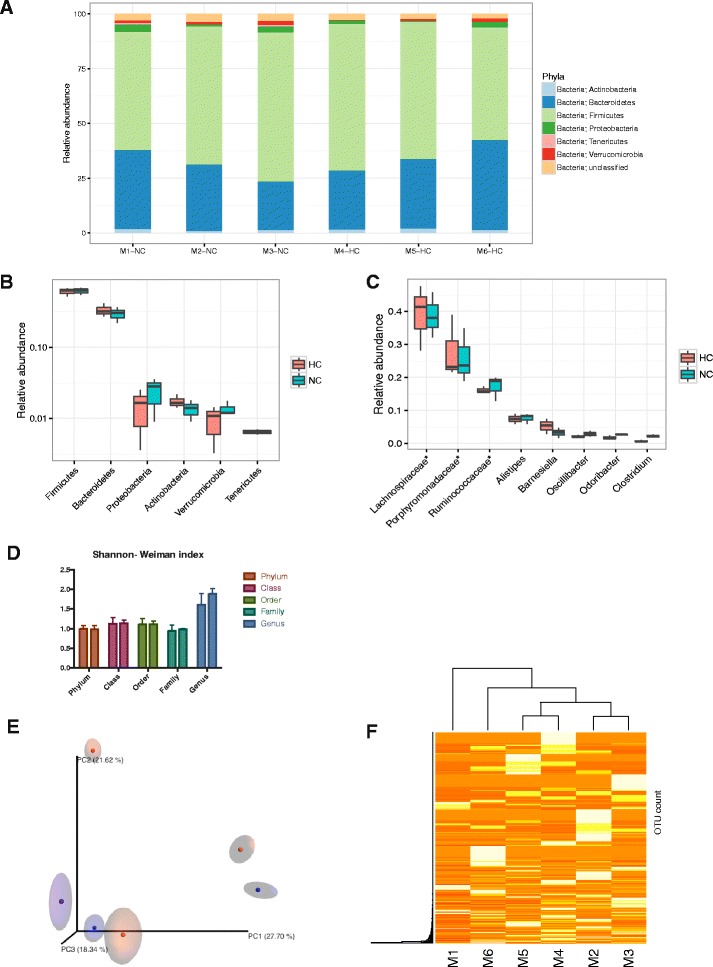
The adaptive host response is not mediated by changes in cecal microbiota abundance. Relative abundance **a** in the individual mice **b** at phylum levels and **c** the 8 most abundant genera taxa. **d** Shannon-Weiman indexes calculated per taxa. **e** Principal component analysis based on unweighted UniFrac distances. **f** Heatmap of the coverage reads of OTUs, limited to 0.01% abundance level. Mice M1-M3 received no cholesterol in the diet (NC), mice M4-M6 were fed high cholesterol diet (HC)

Interestingly, there was no difference in either total or relative abundance of the main microbial phyla in the cecum despite the strong dietary intervention (Fig. 1a-c). Cecal microbial composition of all mice was comprised of *Bacteroidetes* (average 55% across all samples), and *Firmicutes* (27%), followed by *Verrucomicrobia* (14%), *Proteobacteria* (3.7%) and *Actinobacteria* (0.6%) (Fig. 1b). The Shannon-Weiner index, a measure of the α-diversity of the community, also did not differ between our test groups on each taxa level (Fig. 1d, *p* = 0.9, Student t-test). An increased ratio between *Firmicutes* and *Bacteroidetes* has been previously observed in mice fed Western diet [2] and similar data were obtained in response to a diet containing only high fat without added cholesterol [27]. However, in our study feeding the high-cholesterol component of Western diet alone did not affect the *Firmicutes* to *Bacteroidetes* ratio (in either group 0.018 ± 0.004, n.s.), and thus any proposed impact of cholesterol diet on this ratio should be reconsidered. On the other hand, our study does not allow to draw a conclusion about potential effects of an altered *Firmicutes* to *Bacteroidetes* ratio on host cholesterol metabolism. In order to test for similarities between the microbiomes of NC and HC mice we used a PCo analysis based on the unweighted UniFrac values, which showed no clustering of the individual samples (Fig. 1e). The constructed unweighted UniFrac distance matrix, based on all 16S rRNA sequences obtained, confirmed that all microbiomes were at a similar distance to each other (Fig. 1f), reflecting their apparent similarity.

Although, there was an overall uniformity in the distribution of bacterial groups across all samples, one notable exception was the class of *Tenericutes,* and more specifically the genus *Anaeroplasma,* which were only detected in NC mice. Previously, the presence of *Anaeroplasma*, was associated with diet-induced obesity [28]. Another notable exception was the *Firmicutes* genus *Turicibacter*, which was present in all HC mice but not detected in the NC group. An increase in *Turicibacter* has previously been correlated to the amount of cecal butyrate in rats fed a barley-malt based diet with a high fat content [29]. However, it has also been found to decrease in response to high-fat feeding in mice [30] suggesting that *Turicibacter* might be responsive to components in the diet other than fat. According to our results the increase in *Turicibacter* could be related to cholesterol abundance of the diet. This suggests a role of the bacterium for assimilation or sequestration of cholesterol, as it has been previously demonstrated for other colonic bacteria of the *Firmicutes* group [31]. Clearly more research is needed to delineate the pathophysiological importance of these bacteria with low abundance and their potential products for their role in cholesterol metabolism and their potential relevance for the development of cardio-metabolic disease. However, such studies are technically difficult, since the *Turicibacter* genus consists of strict anaerobes with poor survival under laboratory conditions [32].

Dietary cholesterol is structurally similar to plant sterols, which however, are non-absorbable due to differences in the side chain length [33]. A study in hamsters adding 5% plant sterols to the diet revealed a decrease in several taxa in fecal samples, among which *Coriobacteriaceae* and *Erysipelotrichaceae* [34]. By displacing cholesterol from bile-formed micelles in the intestine, plant sterols can increase the total amount of unabsorbed cholesterol in the intestine [35]. Thus plant sterol feeding could be expected to result in increased exposure of intestinal bacteria to cholesterol, similar to our present work. However, differences in the side chain between cholesterol and phytosterols as well as parameters related to specific hamster gut microbial populations may account for the different outcomes of both studies. In humans, plant sterol supplementation seems unrelated to microbiota composition and diversity [36]. Together with our results these findings suggest that plant sterols might have, at least with relevance to rodents, an intrinsic biological activity on intestinal bacteria, which is not shared by cholesterol.

The within-group variation in our study was minimal, likely as a result of our decision to use littermates and thus avoid confounding by kinship. Combined with the depth of the sequencing analysis we therefore expect to have generated sufficiently robust data, a view actually shared by literature using a similar experimental design [9, 37]. In addition, with our chosen approach we provide limited estimates on changes that might occur in bacterial metabolism. A more in-depth approach could include meta-transcriptomics with subsequent evaluation of genetic networks responding to an increased availability of cholesterol. However, based on the absence of any major detectable change with our current experimental design we would not expect that even such an elaborate approach would reveal substantial alterations in critical pathways of bacterial metabolism. Another point not covered by our present study is the potential impact that changes in microbiota induced by factors other than dietary cholesterol might have on cholesterol metabolism either locally in the intestine or systemically. More research involving e.g. conventionalized germ-free mouse models would be required to address this point.

In summary, our results demonstrate that feeding a high cholesterol diet alone does not result in major appreciable alterations in the composition of the intestinal microbiota. This indicates that the substantial adaptive changes in whole body cholesterol homeostasis occur independently from microbial adaptations. We also conclude that the impact of Western diet on the microbiota is exclusively mediated by its high fat content.

## Additional file

Additional file 1: Figure S1. Relative expression of Npc1l1 mRNA in small intestine of mice after 12 weeks on control or 1.25% cholesterol diet (HC). Data is mean ± SEM. Statistical significance was tested with Mann-Whitney U-test. (PDF 185 kb)

## Abbreviations

HC: High cholesterol
LDL: Low density lipoprotein
NC: No added cholesterol
OTU: Operational taxonomic unit
