# Supplementary material for: High-cholesterol diet does not alter gut microbiota composition in mice
Source: Nutr Metab (Lond). 2017 Feb 16;14:15. doi: 10.1186/s12986-017-0170-x (PMC5314487; doi:10.1186/s12986-017-0170-x)
Supplement: Additional file 1: Figure S1. — Relative expression of Npc1l1 mRNA in small intestine of mice after 12 weeks on control or 1.25% cholesterol diet (HC). Data is mean ± SEM. Statistical significance was tested with Mann-Whitney U-test. (PDF 185 kb) [file 12986_2017_170_MOESM1_ESM.pdf]

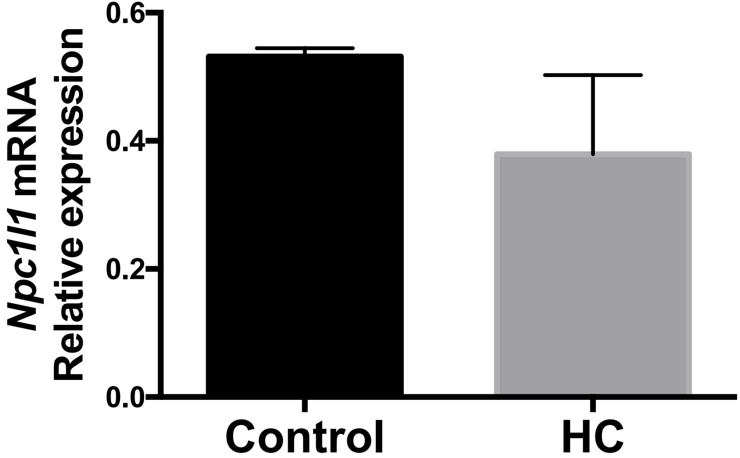

Additional file 1: Figure S1: Relative expression of *Npc1l1* mRNA in small intestine of mice after 12 weeks of control or 1.25% cholesterol diet (HC). Data is mean ± SEM.
Statistical significance was tested with Mann-Whitney U-test.
